# Differences in Electroencephalography Power Levels between Poor and Good Performance in Attentional Tasks

**DOI:** 10.3390/brainsci14060527

**Published:** 2024-05-22

**Authors:** Moemi Matsuo, Takashi Higuchi, Taiyo Ichibakase, Hikaru Suyama, Runa Takahara, Masatoshi Nakamura

**Affiliations:** 1 Faculty of Rehabilitation Sciences, Nishikyushu University, Kanzaki 842-8585, Saga, Japannakamuramas@nisikyu-u.ac.jp (M.N.); 2 Department of Physical Therapy, Osaka University of Human Sciences, Settsu 566-8501, Osaka, Japan; t.higuchi1124@gmail.com

**Keywords:** attentional function, electroencephalography, brain function, neuroimaging, neuroscience, brain injury

## Abstract

Decreased attentional function causes problems in daily life. However, a quick and easy evaluation method of attentional function has not yet been developed. Therefore, we are searching for a method to evaluate attentional function easily and quickly. This study aimed to collect basic data on the features of electroencephalography (EEG) during attention tasks to develop a new method for evaluating attentional function using EEG. Twenty healthy young adults participated; we examined cerebral activity during a Clinical Assessment for Attention using portable EEG devices. The Mann–Whitney U test was performed to assess differences in power levels of EEG during tasks between the low- and high-attention groups. The findings revealed that the high-attention group showed significantly higher EEG power levels in the δ wave of L-temporal and bilateral parietal lobes, as well as in the β and γ waves of the R-occipital lobe, than did the low-attention group during digit-forward, whereas the high-attention group showed significantly higher EEG power levels in the θ wave of R-frontal and the α wave of bilateral frontal lobes during digit-backward. Notably, lower θ, α, and β bands of the right hemisphere found in the low-attention group may be key elements to detect attentional deficit.

## 1. Introduction

Decreased attentional function causes problems in daily life, such as an increase in traffic accidents and an inability to concentrate on work. Attentional function is considered the basis of other cognitive processes [1], and impairment of attentional function can impede rehabilitation interventions. In clinical settings, attentional function is frequently evaluated using assessment tools such as the Clinical Assessment for Attention (CAT), a standardized evaluation method, and the Trail Making Test (TMT). However, the CAT has many test items, and it takes approximately 3 h to evaluate all seven types of attentional functions. In addition, the TMT can only assess a few aspects of attentional function, leading to discrepancies between clinical conditions and assessment results. To date, a method for the quick and easy evaluation of attentional function has not yet been developed. Therefore, our focus remains on searching for a method to evaluate attentional function easily and quickly. If attentional function could be evaluated quickly and easily using a portable EEG, this would lead to a reduction in the time required for evaluating attentional function in clinical settings, ultimately reducing the burden on patients and examiners.

Recently, functional and relevant neuroimaging techniques have become principal instruments in rehabilitation research. These methods can be applied to explore the impacts of cerebral injury or disorder on brain systems associated with cognition and behavior and determine how rehabilitation could alter brain systems, including functional magnetic resonance imaging (fMRI), positron emission tomography (PET), electroencephalography (EEG), magnetoencephalography (MEG), near-infrared spectroscopy (NIRS), and transcranial magnetic stimulation (TMS) [2]. Among these, EEG is a noninvasive, easy-to-use, and relatively inexpensive method for assessing neurophysiological function in a variety of situations, collecting basic data, and reliably assessing cerebral function [3]. Other investigations with greater specificity and sensitivity have largely superseded its value in the diagnosis and evaluation of neurological diseases other than epilepsy. EEG is mostly important for cases of impaired consciousness or altered mental status [4]. Electroencephalography (EEG), the earliest modality for imaging human cortical brain activities, has garnered increasing scientific and clinical interest. For over 40 years, EEG research has been conducted to identify and measure the neurophysiology of attention-deficit/hyperactivity disorder [5], as well as diagnose disorders with unique patterns of atypical resting-state EEG [6]. Brain–computer interfaces (BCIs) are a fast-evolving technology with potential for revolutionizing how humans interact with computers [7]. BCIs quantify brain activity and transform it into commands for computers or other instruments, including EEG, allowing operators to solely guide apparatus and tools with their own thoughts. Therefore, continuously interpreting EEG signals to evaluate brain function is clinically important for developing an attentional function evaluation method. Although EEG has shown promise in measuring attentional function, no method has been developed to easily evaluate attentional function using EEG, resulting in a growing interest in the evaluation of attentional functions using EEG.

A Convolutional Neural Network (CNN), a deep learning method, may be helpful for developing a short and simple method for evaluating attentional functions. A CNN enables the interpretation of EEG signals that are difficult for humans to read. Developing a trained prediction model that accurately predicts attention functions using a CNN and incorporating it into a portable EEG may allow for the easy and quick evaluation of attention functions in the future. Furthermore, a previous study reported that a CNN trained using signals obtained from an EEG could classify four imagined objects with a 60% success rate [8]. In other words, it has been suggested that brain activity related to cognitive functions can be measured using EEG and that cognitive evaluation can be performed by combining the data with a CNN. Specifically, eyes-open EEG alpha attenuation might represent a neural biomarker for risk of attentional impairment [9]. Moreover, the left frontoparietal network, which is related to spatial attention, had stronger connections in the δ, β, and γ bands in the depressive emotion group compared with the healthy control group [10].

Therefore, this study aimed to collect basic EEG feature data during attention tasks to develop a new method for evaluating attentional function. We expect to shorten the evaluation time for attentional function in clinical settings and reduce the burden on patients and examiners, leading to an increase in training time and contribution to rehabilitation interventions. Furthermore, the decline in attentional function not only occurs in subjects with brain damage but can also appear with aging. Therefore, being able to easily evaluate attentional function will be useful for a variety of settings and individuals. Based on these findings, this study may play an important role in resolving these clinical issues. To achieve these objectives, we used a portable EEG.

## 2. Materials and Methods

### 2.1. Participants

Twenty healthy young adults (14 men and 6 women; age: 20.6 ± 1.7 years) participated in this research. All potential participants received extensive explanation about the safety protocols of this study, assuring that their individual identifying information would be kept confidential. Thereafter, the participants gave written informed consent for their inclusion in the study. Additional informed consent was obtained from all participants whose identifiable data were analyzed in this study. The inclusion criteria were patients without a history of major physical disorders, including neurological illnesses, brain injuries, or psychiatric illnesses. The exclusion criteria were patients having a history of one or some of these disorders. No participants had a history of major physical diseases, including neurological disorders, cerebral injuries, or psychiatric impairments. This study was approved by the Ethics Committee of Nishikyushu University (approval no. 23 NMR06; 25 May 2023) and conformed to the principles of the Declaration of Helsinki [11] and its subsequent amendments.

### 2.2. Task

This comprised two tasks from the Japanese Society for Higher Brain Dysfunction. One was the Digit Span Test, which is an auditory memory test. It involves the following two conditions: participants immediately repeat the number sequence read out loud after the examiner (digit-forward) and answer the number sequence in reverse after the examiner (digit-backward). The other test was the Tapping Span test, which is a visual memory test. It also had two conditions, as follows: the examiner pointed out nine squares drawn on a test diagram in order, and participants immediately pointed to them in the same order (tapping forward); then, the participants pointed to them in the reverse order (tapping backward). Each number was in a range of 2–9 digits; there were first and second series. If the participants answered or pointed correctly, they moved on to the next digit. If they misplaced the same digit twice in a row, the test would end. The standard cut-off point of each task was digit-forward—6 (6 digits or less indicated low attention function), digit-backward—4 (4 digits or less referred to a group with low attention function), tapping forward—6 (6 digits or less showed a group with low attention function), and tapping backward—5 (5 digits or less referred to a group with low attention function).

### 2.3. Experimental Protocol

**Figure 1:**
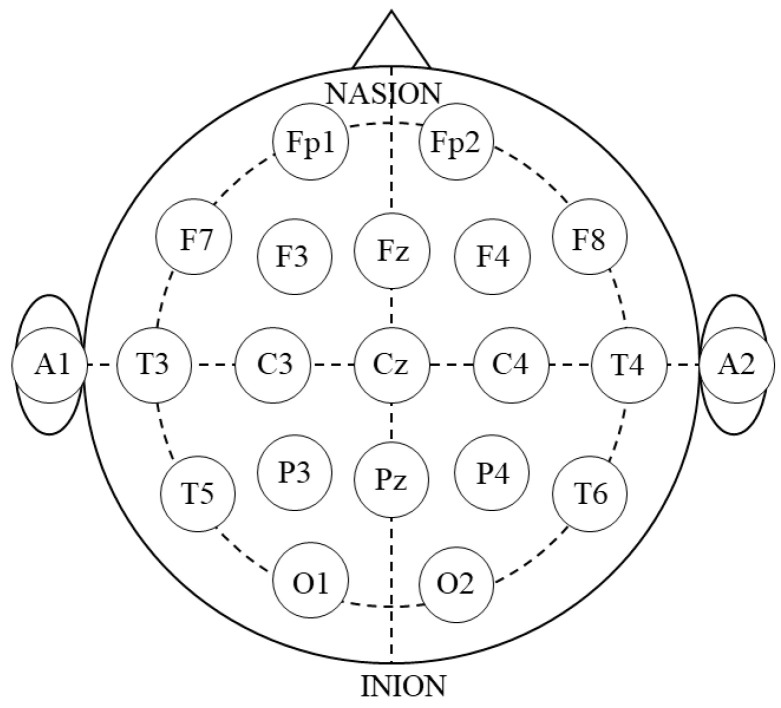
EEG electrode placement. EEG electrodes are positioned based on the international 10–20 EEG placement method. The Fp1, Fp2, F3, Fz, F4, F7, F8, C3, Cz, C4, P3, Pz, P4, O1, O2, T3, T4, T5, and T6 are examined (EEG, electroencephalography; Fp1, left frontal pole; Fp2, right frontal pole; F3, left frontal; Fz, middle frontal; F4, right frontal; F7, left inferior frontal; F8, right inferior frontal; C3, left central; Cz, middle central; C4, right central; P3, left parietal; Pz, middle parietal; P4, right parietal; O1, left occipital; O2, right occipital; T3, left mid temporal; T4, right mid temporal; T5, left posterior temporal; T6, right posterior temporal).

The participants were asked to sit in a quiet room on chairs with backrests; their forearms were placed in a relaxed position on a table. The participants were asked to carry out the tasks without any additional movements, such as head movements, and maintain the same posture during the experiment. EEG measurements were recorded using a Polymate Pro MP6100 (Miyuki Giken, Tokyo, Japan). Prior to electrode introduction, the skin was rinsed with alcohol; the electrodes were affixed to an elastic cap using a holder. According to the international 10–20 EEG placement system, 19 gold-coated active EEG electrodes were positioned at specific cortical locations, as follows: Fp1 (left frontal pole), Fp2 (right frontal pole), F3 (left frontal), Fz (middle frontal), F4 (right frontal), F7 (left inferior frontal), F8 (right inferior frontal), C3 (left central), Cz (middle central), C4 (right central), P3 (left parietal), Pz (middle parietal), P4 (right parietal), O1 (left occipital), O2 (right occipital), T3 (left mid temporal), T4 (right mid temporal), T5 (left posterior temporal), and T6 (right posterior temporal) (Figure 1). EEG measurement at the scalp level represents the aggregate currents of the electrical fields generated by neural activity in cortical neural circuits [12].

### 2.4. Data Analysis

EEG data were collected at a rate of 1000 Hz and filtered within the 1–60 Hz range using a bandpass filter. Artifacts from eye blinks or muscle movements were excluded. Power spectrum analysis was performed using an electromagnetic source estimation data editor (Cortech Solutions, Wilmington, NC, USA). The nine regions of interest (ROIs) were set as L-frontal (Fp1, F3, F7, and Fz), R-frontal (Fp2, F4, F8, and Fz), L-temporal (T3 and T5), R-temporal (T4 and T6), central (C3, C4, and Cz), L-parietal (P3 and Pz), R-parietal (P4 and Pz), L-occipital (O1), and R-occipital (O2). EEG rhythms were categorized into six wave bands according to their frequency ranges, as follows: δ (0–4 Hz), θ (5–8 Hz), α (9–13 Hz), β (14–30 Hz), low-γ waves (31–50 Hz), and high-γ waves (51–70 Hz) based on previous studies [13,14]. The mean power level of each waveband was calculated for each task.

### 2.5. Statistical Analysis

The participants were divided into two groups with cutoff points for each of the following tasks: digit-forward = 6, digit-backward = 4, tapping-forward = 6, and tapping backward = 5. Those scoring at or below the cut-off points were allocated to the low-attention groups, while those with over the cut-off points were allocated to the high-attention groups. The Mann–Whitney U test was used to examine differences in EEG power levels during tasks between groups. IBM SPSS Statistics (version 20.0; IBM Corp., Armonk, NY, USA) was used for the statistical analysis. Statistical significance was set at *p* < 0.05.

## 3. Results

**Figure 2:**
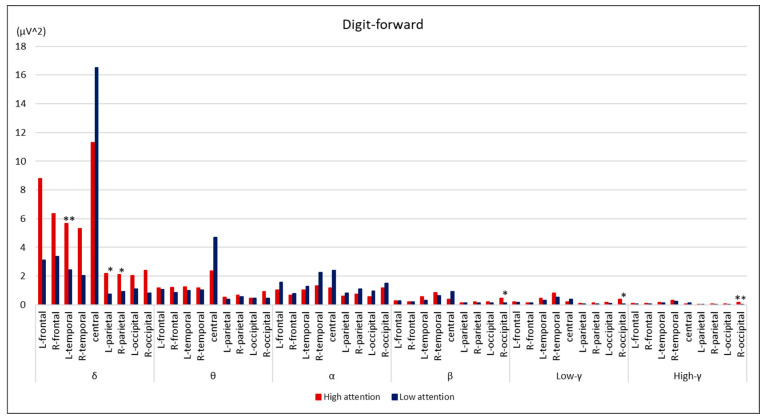
Comparison of EEG power levels between groups during digit-forward task. All data are expressed as average of EEG power levels in each group. The red bars indicate the results in the high-attention group; the blue bars indicate the results in the low-attention group. ** *p* < 0.01, * *p* < 0.05 using Mann–Whitney’s U test.

**Figure 3:**
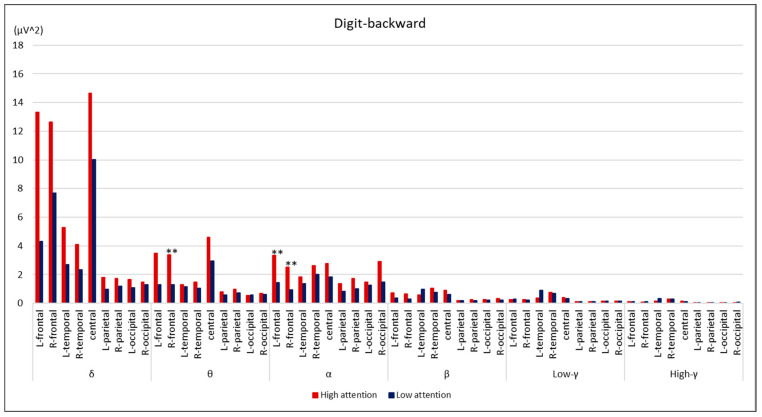
Comparison of EEG power levels between the groups during digit-backward task. All data are expressed as average of EEG power levels in each group. The red bars indicate the results in the high-attention group; the blue bars indicate the results in the low-attention group. ** *p* < 0.01 using Mann–Whitney’s U test.

**Figure 4:**
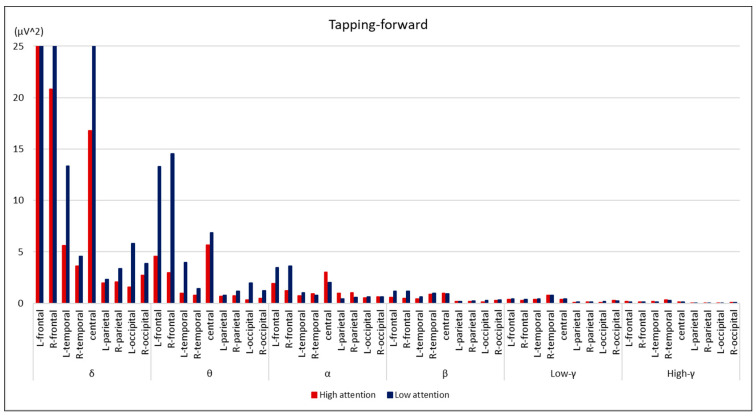
Comparison of EEG power levels between the groups during tapping-forward task. All data are expressed as average of EEG power levels in each group. The red bars indicate the results in the high-attention group; the blue bars indicate the results in the low-attention group.

**Figure 5:**
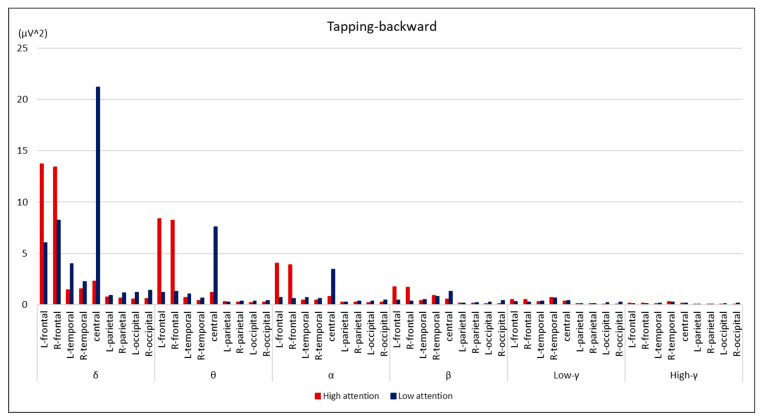
Comparison of EEG power levels between the groups during tapping-backward task. All data are expressed as average of EEG power levels in each group. The red bars indicate the results in the high-attention group; the blue bars indicate the results in the low-attention group.

**Figure 6:**
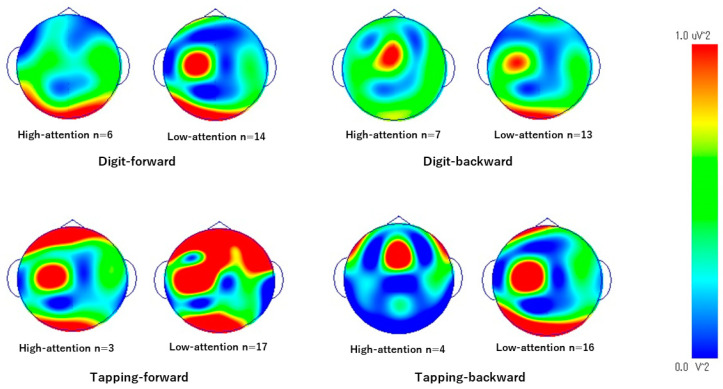
EEG topographic map regarding each group and task. Hotter- and cooler-colored spots indicate higher brainwave power levels and lower brainwave power levels, respectively. Each topographic is shown with 0–70 Hz of the EEG wave band.

Comparisons between the groups are summarized in Figure 2, Figure 3, Figure 4 and Figure 5. During the digit-forward task, the high-attention group showed significantly higher EEG power levels in the δ wave of the L-temporal and bilateral parietal lobes, as well as in the β and low and high γ waves of the R-occipital lobe than the low-attention group. During the digit-backward task, the high-attention group showed significantly higher EEG power levels in the θ wave of the R-frontal and the α wave of the bilateral frontal lobes. No significant differences were observed during the tapping period. The integrated topographic maps of the participants are shown in Figure 6. The results suggest that similar areas, such as the central and occipital areas during the Digit Span and frontal, central, and occipital areas during the Tapping Span, had high EEG power levels.

## 4. Discussion

The essential components of EEG signals comprise brain rhythms in various brain regions, reflecting the activity of these regions. The electrical activity of the cerebral cortex is sent to the scalp through the anatomical structure. Consequently, the measured EEG signals are a combination of source signals from different brain regions that reflect a large amount of spatial location information [15]. In this study, we examined the cerebral activity during CAT using a portable EEG. The low-attention group in the digit-forward task showed significantly lower EEG power levels in the δ wave of L-temporal and bilateral parietal lobes, as well as in the β and γ waves of R-occipital lobe than the high-attention group, whereas the low-attention group in the digit-backward task showed significantly lower EEG power levels in the θ wave of R-frontal and α wave of bilateral frontal lobes; however, no correlations were found during Tapping Span. The Tapping Span involved body movements, which may have caused a variety of EEG data and affected the statistical analysis results, as we could not find consistent results.

In our previous study, the power levels of brain waves varied depending on the type of attention task. During the focused attentional task, the δ wave increased, and the α wave decreased; during the alternating attentional task, the β and γ waves both increased [13]. The θ rhythms have the highest classification performance with visual search [16], cognitive control has been strongly linked to midfrontal θ brain activity [17]. The EEG α rhythm is one of the most salient human brain activity rhythms, modulated by the level of attention and vigilance [18]. A review article suggested that higher α activity improves attention scores [19], and even resting EEG α oscillations are correlated to vigilant attention [20] and visual spatial attention [21]. A previous study investigated the neural patterns in visual attention recognition (i.e., mental arithmetic), where attention states showed less activation than did non-attention states in the prefrontal and occipital areas in the α, β, and θ bands [22]. Another study suggested that α wave as α lateralization plays a causal role in attention tasks [23,24]. Conversely, patients with schizophrenia, who typically exhibit deficits in working memory, showed significantly lower α suppression in the task preparation, memory encoding, maintenance, and retrieval phases, indicating strong α power [25], whereas stronger connections in δ, β, and γ bands were found in the depressive emotion group [10]. These results may imply that attentional network might be modulated due to mental conditions. Clinically, a randomized controlled trial revealed that a lower frontal α power was significantly associated with a higher incidence of postoperative delirium [26], which is usually a reversible disturbance in mental status with a degree of inattention [27]. Another study reported that the results demonstrated a significant correlation between the power spectral density of the EEG β band and students’ academic performance, which relates to their attentional ability [28].

In this study, the lower δ, β, and γ waves during the digit-forward task, which was an easier attentional task, may indicate that the low-attention group could not induce focused or alternating attention. The lower θ and α waves during the digit-backward task, which was more difficult and needed working memory, may indicate that the low-attention group could pay more focused attention. Moreover, the lower EEG power level of the right hemisphere may be representative of lower attentional skill, as the result suggested that δ wave of bilateral parietal, β, γ waves of R-occipital lobe, θ wave of R-frontal, and especially α wave of bilateral frontal lobe were lower in the low-attention group. The role of θ and α power in frontal areas can be biomarkers for both cognitive and physical performances [29]. The frontal poles are brain regions that play a key role in emotional regulation and cognitive abilities [30]. Indeed, a review article suggested that the following EEG biomarkers are most robust; task-induced EEG frontal–midline θ and EEG individual α frequency [31].

These results suggest that we could detect attentional deficits with fewer electrodes focusing on specific brain areas and brain waves such as the θ, α, and β bands (5–30 Hz) of the right hemisphere, as other research has suggested that neural oscillations recorded with ear-EEG could be used to reliably differentiate between levels of cognitive workload and working memory, be integrated into wearable devices in the near future [32], and identify mild cognitive impairments (MCIs) and neural alterations via portable EEG devices designed to capture prefrontal selective attention in combination with behavioral assessments. This approach could potentially augment the use of traditional neuropsychological tests during clinical screening for MCIs [33]. In this study, we compared brain waves between high- and low-attention groups. However, resting-state EEG should also be considered a key point to detect attentional deficits, as previous research has pointed out that those with attention-deficit/hyperactivity disorder have specific electrophysiological conditions [34].

On the other hand, although the task we used in this study was developed to detect attentional dysfunction in patients with neurological disorders, 8 of 20 were consistently detected with low attention function in all tasks, while 12 of 20 were detected with low attention function in some of the tasks in the healthy participants. These might indicate that the tasks are too difficult to evaluate actual patients. We should review the currently used evaluation tools in clinical scenarios and simplify their application using some technologies.

This study has some limitations. First, the participants were all healthy young adults. Therefore, whether our results could be generalized to older patients or those with neurological disabilities remains unclear. Second, the attentional task was limited to the span. Therefore, whether brain waves during other attentional tasks could be comparable to those observed during the span remains vague. Third, we did not distinguish handedness among the participants. This may have affected the functional lateralization of brain processes and the results obtained. Fourth, our sample size was small. Future studies must be conducted with a larger number of participants under various conditions, and brainwaves should be investigated during various attentional tasks.

## 5. Conclusions

Our study identified significant correlations between EEG power levels in specific brain areas and attentional task performance. Notably, lower θ, α, and β bands (5–30 Hz) of the right hemisphere were found in the low-attention group. Thus, the θ, α, and β bands may be key elements to detecting attentional deficit. These results suggest that attentional deficits can be detected with fewer EEG electrodes.

## Data Availability

The data are not publicly available due to the lack of participants’ agreement. The datasets generated and analyzed in this current study are available from the corresponding author upon reasonable request.
